# Little evidence for Fast Mapping (FM) in adults: A review and discussion

**DOI:** 10.1080/17588928.2018.1542376

**Published:** 2018-11-19

**Authors:** Elisa Cooper, Andrea Greve, Richard N. Henson

**Affiliations:** Medical Research Council Cognition and Brain Sciences Unit, University of Cambridge, Cambridge, UK

**Keywords:** Fast Mapping, episodic encoding, hippocampus, learning, memory

## Abstract

Conventional memory theory proposes that the hippocampus is initially responsible for encoding new information, before this responsibility is gradually transferred to the neocortex. Therefore, a report in 2011 by Sharon et al. of hippocampal-independent learning in humans was notable. These authors reported normal learning of new object-name associations under a Fast Mapping (FM) procedure in adults with hippocampal damage, who were amnesic according to more conventional explicit memorisation procedures. FM is an incidental learning paradigm, inspired by vocabulary acquisition in children, which is hypothesised to allow rapid, cortical-based memory formation. In the years since the original report, there has been, understandably, a growing interest in adult FM, not only because of its theoretical importance, but also because of its potential to help rehabilitate individuals with memory problems. We review the FM literature in individuals with amnesia and in healthy adults, using both explicit and implicit memory measures. Contrary to other recent reviews, we conclude that the evidence for FM in adults is weak, and restraint is needed before assuming the phenomenon exists.

## Introduction

A leading theory of memory proposes that new information is quickly acquired by the hippocampus, before the neocortex takes over responsibility for longer-term storage (a process called ‘consolidation’; Mckenzie & Eichenbaum, 2011; Norman & O’Reilly, 2003; Squire & Bayley, 2007). A paper by Sharon, Moscovitch, and Gilboa (2011) therefore caused considerable excitement by describing normal learning in four individuals with amnesia following hippocampal injury. Under standard explicit encoding (EE) instructions, where they were told to remember the names of unknown objects, these individuals showed impaired explicit memory for the names after both 10-minute and one-week delays, as expected. However, under a ‘Fast Mapping’ (FM) learning procedure, which involved incidentally associating the name with the unknown object by answering a question that related it to a simultaneously-presented, semantically-related known object, these individuals performed as well as healthy controls at both delays. This result is remarkable, not only because it suggests rapid cortical learning without consolidation, contrary to conventional theory, but also because of the potential for rehabilitation in individuals with amnesia following hippocampal injury.

**Figure 1.**
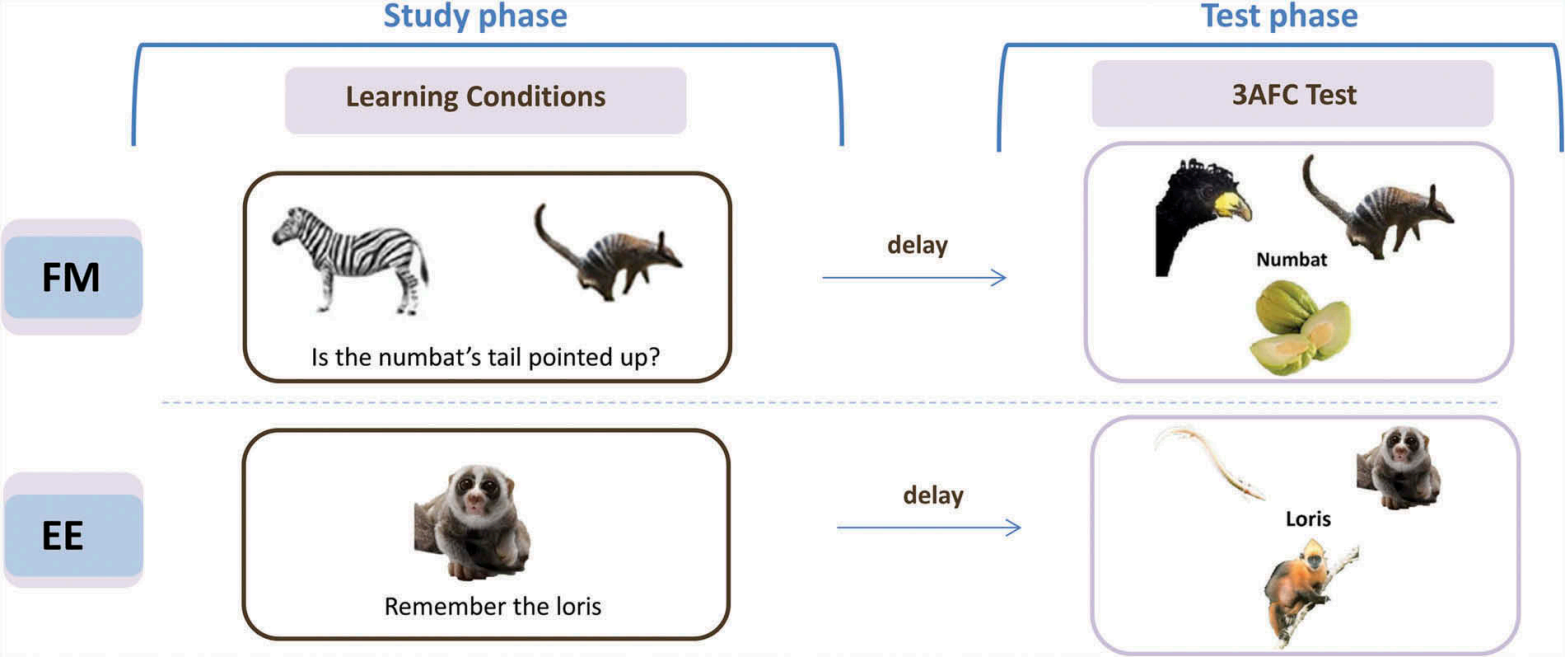
Example of FM and EE learning procedures used in adult populations. Under the Fast Mapping (FM) condition participants should incidentally associate the name with the unknown item while answering the yes/no question. After a delay (e.g., 10-minutes and one-week) there is a surprise memory test (e.g., three alternative force choice (3AFC) recognition). Under the Explicit Encoding (EE) condition participants should intentionally learn the name of the unknown item, and then complete identical delay and test phases. Methodological details differ between experiments. See text for details.

To be more specific, in Sharon et al.’s investigation, four adults with acquired amnesia and a group of matched controls completed two conditions, in which they learned the names of real objects that they did not know (rare animals and fruit). In the FM condition, the picture of the unknown object was presented simultaneously with a known object (semantic referent) and participants answered a yes/no question that required them to infer the name of the unknown object via disjunctive inference (Halberda, 2006) (see Figure 1 for an example of the FM condition). In the EE condition, a single unknown object was presented together with its name, and participants were simply asked to learn the association. For both conditions, recognition memory for the name was assessed 10-minutes and one-week later using a three alternative forced choice (3AFC) test, where individuals had to choose one of three objects that matched a name. Under EE, individuals with hippocampal damage performed worse than controls, as expected, but remarkably, under the FM condition, their memory performance did not differ from controls. Indeed, they showed better memory under the FM than EE condition, whereas controls showed the opposite pattern—a cross-over interaction pattern. The authors concluded that FM allows rapid, cortical learning that emerges in individuals with hippocampal damage. Furthermore, they went on to suggest that this cortical learning requires the anterior temporal lobe (ATL), because an additional group of two individuals with ATL damage (but not hippocampal damage) failed to show the same FM benefit. Because the ATL has been associated with semantic knowledge (Patterson, Nestor, & Rogers, 2007), these results suggest that learning under FM results from rapid semantic integration of new knowledge.

Given this potentially ground-breaking result, a great deal of interest was generated from Sharon et al.’s study. In the seven years since publication, there have been attempts to replicate the FM advantage. There have been investigations in other individuals with amnesia following hippocampal injury, as well as investigations of FM learning in young and older healthy adults with explicit memory tests, neuroimaging measures, and more recently, implicit memory tests. Here we review this literature, but first we address the origin of the FM concept.

## FM in infant word learning

The FM procedure developed from ideas about how infants rapidly acquire vocabulary from relatively few learning exposures, often termed ‘fast mapping’ (for reviews, see Carey, 2010; Samuelson & McMurray, 2017; Swingley, 2010; see also Markson & Bloom, 1997; for a report of fast mapping extending beyond the word learning domain). There is no doubt that infants must match new labels with new objects in order to acquire a large vocabulary over relatively few years (Halberda, 2006), during which time the hippocampus is also rapidly developing (Utsunomiya, Takano, Okazaki, & Mitsudome, 1999). In one of the initial studies, which involved inferring the label ‘chromium’ for an olive-colour, Carey and Bartlett (1978) define ‘fast mapping’ as a special *process* whereby new labels are learned by correctly identifying a new term and its referent, while gaining aditional knowledge, e.g., partial semantic information (see also Carey, 1978). During their vocabulary acquisition, infants are able to recognise a new phonological form, and establish partial lexical, syntactic and semantic knowledge (Bloom, 2001; Bloom & Markson, 1998; Carey, 1978, 2010; Heibeck & Markman, 1987; Katz, Baker, & Macnamara, 2008). This occurs even when associations must be inferred from relating continuous adult speech to the infant’s environment (i.e, without explicit instruction about the new name).

Key to infant fast mapping is selecting the referent: deducing what item is being referenced against a background of already known objects in the environment (Bloom & Markson, 1998; Carey & Bartlett, 1978; Heibeck & Markman, 1987; Markman, 1990; Spiegel & Halberda, 2011). How infants accomplish referent selection is debated, but one possible mechanism, which is used by children and adults, is disjunctive inference (Halberda, 2006). Note that disjunctive inference, or the ‘process of elimination’, does not necessarily involve semantic information. Though infants might gain information about the referent’s meaning (Carey, 1978), semantic relatedness between known and referent items is not an established requirement for infant fast mapping paradigms (e.g., the paradigms of Axelsson & Horst, 2014; Horst & Samuelson, 2008; Spiegel & Halberda, 2011). Referent selection can be influenced by factors external to the known item, such as cues from the speaker (Baldwin, 1993), and by features of the known item, such as salience (Pomper & Saffran, 2018) and co-occurrence with the unknown item (Axelsson & Horst, 2014). Smith and Yu (2008) demonstrated that referent selection can occur without known items at all; a referent can be deduced from cross-situational mapping over multiple trials. Therefore, while semantic information may or may not be key to infant fast mapping, referent selection (through some form of inference) appears manditory.

The partial information gained under fast mapping may not result in long-term retention: it may be forgotten, especially without further exposure (e.g., Carey & Bartlett, 1978). Therefore, a slower process with repeated experiences, ‘extended mapping’, is believed key for retention of the initial partial label knowledge, and its evolution into a more complete and flexible representation of the word and concept (Carey, 2010; Carey & Bartlett, 1978; Swingley, 2010). Initial studies (Carey, 1978; Carey & Bartlett, 1978) reported that infants can retain information about the new label for at least a week, even though infants may not produce the label during that time. However, four studies by Horst and Samuelson (2008) reported no evidence for retention after successful fast mapping label-object matching; long-term retention was only evident when the experimenter explicitly held the unknown object and named it again.

This question of length and nature of retention after fast mapping led some researchers to speculate whether fast mapping in infancy is, in fact, any different from normal learning processes (Alishahi, Fazly, & Stevenson, 2008; Bion, Borovsky, & Fernald, 2013; Vlach & Sandhofer, 2012). Bion et al. (2013) report that access and use of label knowledge improved over time and re-exposure. This suggests that name acquisition is a prolonged learning process in which representations are accrued throughout infant and toddler years. This led to the suggestion that fast mapping is simply referent-matching and not word learning per se (though see Spiegel & Halberda, 2011). Additionally, forgetting rates under fast mapping in infants and adults were reported as similar to those for other, standard memory tasks, suggesting that standard memory consolidation processes are required for long-term retention beyond initial mapping (Vlach & Sandhofer, 2012). A recent computational model accounted for infant fast mapping results through bootstrapping (Alishahi et al., 2008), in which new knowledge leverages on existing knowledge, as in normal memory processes. Thus it is currently unclear whether fast mapping in infancy is the start of standard vocabulary learning (e.g., Bion et al., 2013), or a special mechanism gathering partial lexical information that requires extended mapping (e.g., Carey, 2010). In short, whether fast mapping in infants is a special process distinct from standard memory mechanisms remains open to debate.

## FM in additional amnestic groups

The FM paradigm developed by Sharon et al. (2011) for adults was designed to mimic the important features of infant learning. More specifically, it was assumed that FM is underpinned by i) incidental learning, ii) semantic features shared between unknown and known objects and iii) a disjunctive semantic inference between a known and an unknown object. However, since this initial study, results from further studies attempting to replicate a FM learning advantage in amnestic populations have been inconsistent. In a near-replication (Table 1), Smith et al. (2014) found no evidence of a FM benefit across two experiments involving seven amnestic individuals with hippocampal damage. Patients’ memory performance was 1) worse than controls on both FM and EE conditions at both 10-minute and one-week delays, and 2) equally badly under FM as under EE. In other words, these authors failed to replicate the critical interaction between FM versus EE and patients versus controls, whereby the memory impairment in patients is reduced for FM versus EE, (i.e, patients’ memory performance differs from controls under EE but not FM). They concluded that FM learning did not behave any differently from standard EE learning.

**Table 1.** Summary of fast mapping studies in adult populations presented in this review.

**Reference**	**Exp. number**	**Groups**	**Is there an interaction between Group and FM versus EE ?**	**Differences between FM and EE**	**Measure**	**Measure**omment
Sharon et al. (2011)	1	H, N = 6 *Versus* CON, N = 15 ATL N = 2 *Versus* CON, N = 15, as above.	Yes No	H: FM> EE CON: EE> FM ATL: EE> FM *** = *** CON: EE> FM	3AFC 10-minutes and 1-week	Original FM paradigm in adults FM Incidental = Yes FM Ref. present = Yes FM Infer. Feature question = Yes No. of unknown stim = 16
Smith, Urgolites, Hopkins, and Squire (2014)	1 2	H, N = 7 *Versus* CON, N = 11 H, N = 7, as Exp 1 *Versus* CON, N = 9	No No	H: EE = FM = BL ***<*** CON: EE> FM H: EE> FM ***<*** CON: EE> FM	3AFC 10-minutes and 1-week	Near-meth. repl. FM Incidental = Yes FM Ref. present = Yes FM Infer. Feature question = Yes Exp. 1 replicated method of Sharon et al. Added: categorisation test No. of unknown stim = Exp. 1 like Sharon et al. Exp. 2 = 24, shown 4x
Warren and Duff (2014)	1	H, N = 4 H-M, N = 6 *Versus* CON, N = 10	No	H: EE = FM = Chance, BL HL **< **CONT H-M: EE = FM > Chance *** = *** CON: EE = FM > Chance EE = FM	3AFC 30-minutes Eye tracking	Altered FM procedure FM Incidental = Yes* (*consent form used “name learning”) FM Ref. present = Yes FM Infer. Feature question = No Added: familiarisation, click on item at study, extensive memory testing phase No. of unknown stim = 24
Warren, Tranel, and Duff (2016)	1	TL, N = 6 *Versus* CON, N = 6	No	TL: EE = FM = Chance ***<*** CONT EE = FM > Chance EE = FM	3AFC 30-minutes Eye Tracking	Altered FM procedure As Warren and Duff (2014) above No. of unknown stim = 24
Merhav, Karni, and Gilboa (2014)	2	H, N = 3 *Versus* CON, N = 28	Yes	Non-interfered pairs H: EE (= chance) < CON: EE H: FM = CON: FM	4AFC 24-hours Non-interfered pairs	Near-meth. repl. Investigated interference effects FM Incidental = Yes FM Ref. present = Yes FM Infer. Feature question = Yes Added: re-paired subsets of objects with a 2^nd^ new name to create “interfered pairs” No. of unknown stim = 20
*As above.*	1	YCON, N = 100	N/A	Non-interfered pairs EE > FM	4AFC 24-hours Non-interfered pairs	As above. No. of unknown stim = 24
Merhav, Karni, and Gilboa (2015)	1	YCON, N = 32	N/A	EE > FM FM ATL activation > EE ATL activation FM HP activation = EE HP activation	4AFC 30-mins & 24-hours fMRI at retrieval	Near-meth. repl. FM Incidental = Yes FM Ref. present = Yes FM Infer. Feature question = Yes Half of items studied 24- hours before test, other half studied 30-mins before test No. of unknown stim = 100
Atir-Sharon, Gilboa, Hazan, Koilis, and Manevitz (2015)	1	YCON, N = 40	N/A	EE > FM FM ATL MVPA decoding > chance EE ATL MVPA decoding = chance	4AFC 30-mins & 24-hours fMRI at study	Near-meth. repl. FM Incidental = Yes FM Ref. present = Yes FM Infer. Feature question = Yes No. of unknown stim = 62
Himmer, Müller, Gais, and Schönauer (2017)	1	YCON, N = 76	N/A	EE Wake-I > EE Wake-12 EE Sleep-I = EE Sleep-12 FM Wake-I = FM wake-12 FM Sleep-I = FM Sleep-12	3AFC Immediate (I) 12-hours (12)	Near-meth. repl. Investigating learning, sleep and consolidation FM Incidental = Yes FM Ref. present = Yes FM Infer. Feature question = Yes Wake groups remained awake between I and 12, Sleep groups slept No. of unknown stim = 40
Greve, Cooper, and Henson (2014)	1	OCON, N = 24 *Versus* YCON, N = 24	No	OCON H-volume reduced due to healthy aging OCON: EE > FM ***<*** YCON: EE > FM Positive correlations between memory under EE *and* FM and H- volume No significant correlations with ATL volume	3AFC 10-minutes and 1-week Grey-Matter volumetrics	Near-meth. repl. FM Incidental = Yes FM Ref. present = Yes FM Infer. Feature question = Yes No. of unknown stim = 24
*Cooper et al. (under review)*	1–3	YCON, N = 24 YCON, N = 24 OCON, N = 24	No	YCON > OCON FM-IR > FM-I = FM-R > FM	3AFC 10-minutes	Memory performance under original FM and FM-variants. FM: Original FM task FM-I: removal of feature inference FM-R: removal of ref. FM-IR: removal of both No. of unknown stim = 24
Coutanche and Thompson-Schill (2014)	1–2	YCON, N = 50 YCON, N = 60	N/A	EE > FM FM learning > EE learning FM RT target > FM RT control words EE RT target> EE RT control words	3AFC 10-minutes Natural/Man-made implicit task 10-minutes	Near-meth. repl. FM Incidental = Yes FM Ref. present = Yes FM Infer. Feature question = Yes Added implicit RT measure in a natural/man-made task Exp 1 added a semantic priming test Also include “IE” condition with removal of ref. No. of unknown stim = 16
Coutanche and Koch (2017)	1	YCON, N = 90	N/A	EE > FM FM RT target > FM RT control words *Only with* atypical known items in FM & participants with high semantic trait score	3AFC 10-minutes Natural/Man-made implicit task 10-minutes	Investigated boundaries of above implicit effect FM Incidental = Yes FM Ref. present = Yes* (*Manipulated) FM Infer. Feature question = Yes Measuring individual traits Manipulating Ref. item No. of unknown stim = 16

**ATL **= Individuals with anterior temporal lobe injury; **H** = Individuals with acquired amnesia and hippocampal injury; **H-M** = Individuals with mild acquired amnesia and hippocampal injury; **TL** = individuals with Temporal Lobectomies; **CON** = matched healthy controls; **YCON **= young healthy adults; **OCON **= Older healthy adults; **BL **= Baseline; performed test without study; **3AFC **= three alternative forced choice memory test; **Near-meth. repl. **= Near-methodological replication of Sharon et al. 2011’s paradigm, including similar FM procedure; **No. of unknown stim** = Total number (No.) of unknown stimuli to be learned under FM only, e.g., not including known catch trials, splitting of FM stimuli for FM delay conditions, or unknown stimuli for other learning conditions; **FM Incidental** = Is the component of incidental learning present in the FM procedure?; **FM Ref. present** = Is the component of related known semantic referent (Ref.) present in the FM procedure?; **FM Infer. Feature question** = Is the component of asking a disjunctive inference (Inf.) question based on feature information present in the FM procedure?; **N** = total number volunteers, prior to data exclusion, and excluding within or between volunteer design information; **Exp**. = Experiment; **Measure** = measures of memory analysed (note: not exhaustive). See text for additional details.

Using a different type of FM procedure, two studies by Warren and colleagues (Warren & Duff, 2014; Warren et al., 2016) also failed to find a benefit of FM over EE for individuals with memory problems. In the study phase of their procedure, participants clicked a mouse cursor on the picture of the unknown item. Under FM, participants saw a known and an unknown item presented simultaneously and a sentence ‘Click on the Numbat’, for example. Under EE, an unknown item was presented with the sentence ‘This is a Mangosteen’, for example. Therefore like Sharon et al. (2011), this FM task involved the presence of other, known objects, but unlike Sharon et al. (2011), the inference was not a question and did not make reference to any features of the objects. These FM and EE study conditions were preceded by a familiarity phase that asked participants about their familiarity with the unknown objects (so that pre-experimentally known objects could be excluded in analysis), and succeeded by a free recall test that was performed immediately, and again after a 30-minute delay. This was followed by a 3AFC test, and then a final cued recall test.

In Warren and Duff (2014), the results showed that four severely amnestic individuals remembered significantly less than healthy older controls under both FM and EE on all tests, and 3AFC performance did not differ from chance. An additional group of six mildly amnestic individuals also failed to show better learning under FM than EE, in all tests. Additional eye-tracking data failed to show differences between EE and FM conditions. In their later investigation, Warren et al. (2016) showed similar results with six individuals with temporal lobectomies: On all tests, their performance under FM and EE was near chance and was significantly worse than healthy controls. However, neither investigation showed the usual pattern of better memory under EE than FM in healthy controls. Rather, the comparable levels of FM and EE performance might be due to the fact that the familiarity phase was run before rather than after the main study/test phases and extensive testing phase. Indeed, Atir-Sharon and colleagues (Atir-Sharon et al., 2015) suggested that Warren and Duff (2014, 2016) protocol deviation may have affected the cortical mapping (FM) process, explaining the lack of benefit for patients.

To our knowledge there has been only one study, from the original group, that replicated the FM benefit in patients (Experiment 2 in Merhav et al., 2014). This study used an additional manipulation of interference effects. In the study phase, these authors re-paired a subset of the unfamiliar objects with new names five-minutes after initial learning, to introduce associative interference, before testing memory 24 hours later. For the non-interfered object-name pairs, the authors found that memory performance in three patients with hippocampal damage (different individuals from those in Sharon et al., 2011) was impaired relative to controls in the EE condition, but not the FM condition, where they performed similarly to controls; a pattern of results in keeping with Sharon et al. (2011). For the interfered pairs however, the patients were reduced to chance levels for FM (and remained at chance for EE), for both the initially-paired and re-paired names, whereas controls were also at chance for FM, but remained above chance for EE. In other words, interference removed any advantage of FM over EE for the patients. The authors made an interesting suggestion that FM is very sensitive to interference, which may explain why other studies did not find the FM advantage, for example, if those studies used too many study pairs (or additional stimuli like those used in the categorical judgement task inserted by Smith et al., 2014).^1^ 1Note that this interference account is compounded by the fact that the FM condition is often run before the EE condition, in order to minimise intentional encoding strategies in the FM condition. This may increase proactive interference for the EE condition, which, if patients are particularly prone to interference in general (as suggested by other studies, e.g., Warrington & Weiskrantz, 1974; Winocur & Weiskrantz, 1976), could explain why their FM performance exceeds their EE performance. This proposal is in line with reports from the infancy FM literature, where competition from within and across trials, impairs retention (Horst & Samuelson, 2008).

### FM in an amnestic group in our own lab

In our own lab, we have also investigated FM learning in individuals with acquired amnesia following hippocampal lesions. We used procedures identical to Greve et al. (2014), which are a near-methodological replication of Sharon et al. (2011) and illustrated in Figure 1. The FM study-test phases were completed prior to EE study-test phases, with a 10-minute delay between phases, and between conditions, during which participants completed non-verbal assessments. To date, three individuals with acquired amnesia and injury that included the hippocampus in all cases (patients P2, P5 and P6 described in Henson et al., 2016) have performed the FM and EE tasks. Healthy control data were from the older group in Greve et al. (2014). We summarise information about the individuals with amnesia here, for full details see Henson et al. (2016).

P2 was a 39-year-old male with a 20 year history of amnesia following carbon monoxide poisoning, and showed marked impairments on neuropsychological tests of verbal and visuospatial memory with no indication of impairment in other domains. Comparisons of subcortical regions to age- and sex-matched controls showed significantly less grey-matter volume in the pallidum and hippocampus only. He performed worse under FM (.46) than EE (.67).

P5 was a 57-year-old female with a six year history of amnesia following limbic encephalitis and showed impairment on tests of verbal and visuospatial memory, and forward digit span. Notably, she had a high pre-morbid IQ and often employed complex strategies to help her perform on memory tasks. Grey-matter analysis showed reduced volume in hippocampus only. She performed marginally better under FM than EE (accuracies of .54 versus .50, respectively).

P6 was a 62-year-old male with a history of amnesia following limbic encephalitis, 14 and four years prior to testing. He showed impaired performance on verbal memory tests and backwards digit span, and reported mild anxiety and depression, though there was no indication of impairment of cognitive functioning in other domains. Grey-matter analysis showed reduced volume in the hippocampus, amygdala, parahippocampus and entorhinal areas. He performed worse under FM (.38) than EE (.42).

When compared on average with the healthy older controls in Greve et al. (aged 55–79), individuals with hippocampal lesions performed numerically worse under EE (.53 versus .76), as expected, but also performed worse under FM (.46 versus .54). While one of our three patients (P5) did perform numerically better under FM than EE, this was also true for three of our 24 older healthy controls. This atypical pattern could reflect different strategies, or could just be random measurement noise. We are therefore yet to find convincing evidence that the FM procedure recovers learning in our own sample of individuals with hippocampal injury.

In general, the FM benefit to individuals with memory problems and hippocampal damage has proved difficult to replicate, though it remains possible that the precise boundary conditions (e.g., level of interference) are vital. Nonetheless, other research has investigated learning under FM in healthy adults, using both explicit memory measures like in the above patient studies, and also novel implicit measures of lexical/semantic integration.

## FM in healthy groups with explicit measures

A consistent finding in the control groups of the above patient studies is that memory performance is better under the EE than FM condition. One possibility is that qualitatively different encoding processes occur in EE and FM conditions. For example, episodic encoding, possibly supported by the hippocampus, might underlie performance in the EE condition, while semantic integration, possibly supported by ATL, might underlie performance in the FM condition, and those processes occurring in the EE condition are just more effective (at least for tests of explicit memory; see subsequent section). An additional possibility is that episodic encoding processes also occur in the FM condition (provided the hippocampus is intact)—i.e, explicit memories are automatically formed even though the FM task is incidental—but these encoding processes are simply less effective than when applied intentionally in the EE condition. Indeed, a further possibility is that episodic encoding processes actually inhibit or mask semantic integration (fast mapping) processes.

Nonetheless, to the extent that some processes occur in the FM task that do not occur in the EE task (e.g., rapid semantic integration)—and assuming these are not completely masked by concurrent episodic encoding processes that might co-occur in the FM task—performance on the FM and EE tasks in healthy controls should be functionally dissociable (even if overall explicit memory performance in the FM condition never exceeds that in the EE condition). One way to dissociate them has already been mentioned above, namely the degree of interference that results when some objects are repaired with new names.

Interference effects were directly investigated in young healthy people in Experiment 1 of Merhav et al. (2014), in which interfering pairs were presented either five-minutes after initial FM or EE learning (early interference), or 22-hours after learning (delayed interference). While memory performance under FM was generally worse than under EE, FM performance was more sensitive to interference than EE performance, particularly in the delayed condition. This is consistent with the patient data in suggesting that FM learning involves different mechanisms than EE learning. However, this finding could also simply reflect a general tendency for greater interference for information that is learned less well, i.e, in FM than EE conditions.

Another way in which processes underlying FM and EE conditions might dissociate is in terms of the patterns of neural activity elicited during encoding or retrieval. Merhav et al. (2015) tested this by using functional magnetic resonance imaging (fMRI) during the memory test phase in young healthy individuals. The study phase was similar to the original Sharon et al. (2011) paper, but with participants assigned to either an FM or EE learning group. Furthermore, half of the study items were studied on one day and the other half on the next day, and then scanned with 4AFC, 30-minutes after the second list on Day 2. As with other healthy samples, memory performance was worse under FM than EE at both time points.^2^ 2Interestingly, the total list length of 100 items was longer than used in previous studies. This was presumably in order to maximise fMRI power, even though it is possible that this increased the interference in the FM condition, as warned by Merhav et al., 2014. Nonetheless, fMRI data showed that items remembered correctly produced differential activation across FM and EE groups, in several brain regions, including greater activation for FM than EE in the ATL. This is consistent with the Sharon et al. (2011) findings of impaired FM learning after ATL lesions, and with more general claims that FM rapidly integrates information into semantic memory. Further fMRI analysis revealed some differences between the patterns of covariation across voxels between the FM and EE conditions. No difference was found in the hippocampus however, consistent with the above possibility that episodic encoding processes still occur during the FM condition.

A similar neural dissociation was reported by another fMRI experiment on healthy young adults (Atir-Sharon et al., 2015), which entailed scanning during the study phase rather than test phase. More specifically, these authors used multi-voxel pattern analysis (MVPA) to decode where patterns of activity across voxels during FM or EE study phases predicted subsequent memory in the test phase (i.e, correct versus incorrect memory performance). As in other studies, memory was better under EE than FM. Above-chance classification of subsequent memory was found in ATL during the study phase for the FM condition, but not for the EE condition, whereas classification in the hippocampus was above chance in both FM and EE conditions. Thus, like the Merhav et al. (2015) study, the fMRI results suggested additional neural activity occurring in the ATL in the FM condition relative to EE condition. Nonetheless, neither study formally established a qualitative difference in brain activity that is sufficient to claim that the cognitive processes differ (Henson, 2006).

It has also been claimed that FM and EE performance dissociates as a function of sleep. Himmer et al. (2017) compared FM and EE learning performance when participants did, or did not, sleep between study and test phases. The Wake and Sleep groups completed either FM or EE learning procedures, and then their memory was tested with 3AFC both immediately and 12 hours later, where the second memory test followed either sleep or wakefulness, respectively. The authors reported a significant difference between the two memory delays in the EE wake group (i.e, worse performance after 12 hours of wakefulness) that was not present in EE sleep group, nor in the FM sleep or wake groups. From this result, they concluded that consolidation during sleep is important for EE, but not for FM, which is more rapidly consolidated. One potential concern here is that immediate test performance in the EE wake group was higher than in the other three groups (even before sleep in the EE sleep group), which may have been an artefactual reason why the forgetting after 12 hours appeared selective to that group. Additionally their hypothesis about the role of consolidation during sleep also seems difficult to reconcile with the several studies that have not reported any interaction between FM versus EE across 10-minutes versus 1-week retention intervals (Greve et al., 2014; Merhav et al., 2015; Sharon et al., 2011; Smith et al., 2014).

It is possible that FM and EE performance dissociate as a function of age, particularly given that the hippocampus (and episodic encoding) appears to be particularly affected by ageing (Greve et al., 2014). Indeed, it is possible that under this FM task older people might behave like amnesic patients with mild hippocampal damage and show a FM advantage like in Sharon et al. (2011). However, this was not supported in a study by Greve et al. (2014). They failed to find an interaction between FM versus EE memory performance and Young versus Older groups, even though MRI confirmed hippocampal volume loss in the Older group. FM and EE paradigms were similar to Sharon et al. (2011), but with ‘unknown’ stimuli normed for a UK population (e.g., unknown item ‘rhubarb’ from the original study, which was conducted in Israel, is known in the UK). Though the Older group showed worse memory overall, the two groups showed a comparably-sized reduction in 3AFC memory performance under FM than EE, at both short (10-minutes) and long (1-week) retention intervals. Moreover, further analyses using structural MRI data revealed significant positive correlations between hippocampal volumes and 3AFC performance under EE, as expected, but also under FM, while no correlation was found between ATL volume and either EE or FM performance. Contrary to the above fMRI studies, these structural MRI correlations suggest that both learning tasks are underpinned by the same neural substrate, i.e, hippocampus. Nonetheless, it remains possible that structural measures of ATL volume are too noisy to reveal a correlation with FM performance, or even that ATL volume is irrelevant compared to functional activity in ATL (as measured with fMRI). Furthermore, it is possible that the FM behavioural benefit only emerges when the hippocampus is more severely damaged (e.g., by a lesion) than occurs during normal ageing.

Other studies have attempted to dissect the critical elements of the adult FM learning procedure. As mentioned above, two components proposed to support adult fast mapping are: 1) the provision of a known semantic referent and 2) the requirement for the new association to be inferred (even if the development literature does not consider a semantic referent, as reviewed above). For example, in their Experiment 2, Coutanche and Thompson-Schill (2014) added a condition (their ‘IE’ condition) in which the task was identical to Sharon et al.’s (2011) FM task, except there was no semantic referent. In healthy young adults, they found that explicit memory was not significantly impaired by removal of the referent.^3^ 3The main focus of this study was actually on implicit measures of memory, which we discuss later. With their implicit measure, there was no evidence of learning in their IE condition, even though there was in their ‘standard’ FM condition. In a more recent investigation, Cooper, Greve, and Henson (*under review*), both proposed components of the FM condition were systematically removed across procedural variants of the FM study phase. Over three study-test cycles, participants in each experiment performed: i) the ‘standard’ FM task (used by Sharon et al., 2011), ii) a variant that removed one component (either the semantic referent, like Coutanche and Thompson-Schill’s IE condition, or the semantic inference), and iii) a variant that removed both components. Memory was tested using 3AFC after a 10-minute delay, before the next condition was run (condition order was counterbalanced). Experiment 1 provided no evidence that removing the known semantic referent harmed memory performance, while Experiment 2 provided no evidence that removing the inferential question harmed memory. Experiment 3 replicated Experiment 2, but with healthy older individuals, based on the hypothesis (like in Greve et al., 2014) that the original FM procedure might particularly benefit those with reduced hippocampal volume. However, there was no evidence that eliminating the inferential question impaired memory more in the older than the young group. In fact, a combined data analysis showed that the general pattern was the opposite to what would be expected if all FM components are key for learning: the simplest FM variant learning procedure with the fewest components (i.e, the variant with neither a semantic referent nor semantic inference) produced the best, rather than worst, memory performance. Cooper et al. hypothesized that, at least in healthy adults using explicit measures, the additional components of the FM task increase cognitive demands during the study phase, which harms rather than helps memory. In other words, there is, as of yet, no evidence that the components of the original FM procedure that have been proposed to be essential for fast mapping offer any benefit to explicit memory in healthy adults. Again however, it remains possible that these components do help fast mapping, but hinder the episodic encoding that, as raised above, may also occur in the FM task (in people with intact hippocampi), such that the net effect is an impairment of explicit memory.

Thus the evidence for functional dissociations in explicit memory between FM and EE conditions in healthy participants is mixed: fMRI provides some support for different brain regions being involved during encoding and retrieval. Additionally FM appears more sensitive to interference, but less sensitive to intervening sleep, than EE. However, the role of sleep remains unclear, particularly given that few differences between immediate and delayed tests have been reported by other studies (where the delayed condition entails at least one night of sleep). Other studies have failed to find a dissociation between FM and EE as a function of age, that might resemble the dissociation reported by Sharon et al. (2011) for hippocampal patients versus controls (i.e, memory being relatively less impaired by age under FM than EE). Furthermore, attempts to isolate the critical components of the FM task have not revealed any benefit of either a semantic referent or a semantic inference. However, the potential masking of explicit memory performance in the FM task by simultaneous, incidental, hippocampally-mediated episodic encoding might potentially reduce the ability to dissociate the two tasks in healthy adults. One way to bypass this problem is to test memory performance on implicit measures, for which episodic encoding is less likely to contribute.

## FM in healthy groups with implicit measures

There are claims that the neural mechanisms that support implicit memory, like priming, do not involve the hippocampus, even for new associations like object-names (e.g., Goshen-Gottstein, Moscovitch, & Melo, 2000). Implicit measures may therefore offer a way to minimise contamination of FM memory performance by episodic encoding. Implicit measures should also produce FM performance in controls similar to that in patients with hippocampal damage, though we are not aware of any patient FM studies using implicit measures.

Implicit measures in FM have been used in studies with healthy adults as reported by Coutanche and Thompson-Schill (2014). These authors found better learning in FM than EE conditions in young adults, using similar learning tasks as the studies above, but using a different, implicit test of memory for the new names. These authors used contrived lexical neighbours (e.g., ‘ganaxy’) of hermit words, which are words with no lexical neighbours (e.g., ‘galaxy’; Bowers, Davis, & Hanley, 2005), and used these as the ‘names’ of unknown animals. Immediately following either FM or EE study phases, where these neighbours were the names of the animals, participants completed the usual explicit memory tests of free recall and 3AFC. The authors reported the typical pattern, i.e, better memory after EE than FM learning. Additionally the authors also measured reaction times (RTs) to make a natural/man-made decision about hermit words. Here they found longer RTs for hermit words for which a neighbour had been studied (as a novel object name) than hermit words without a studied neighbour, but only after FM learning. We expand on theoretical interpretation of this finding below.

Prior research has shown that neighbourhood density affects word recognition: The greater a word’s lexical neighbourhood, the greater the competition during word recognition, resulting in slower responses to that word (Andrews, 1996; Bowers et al., 2005; Davis & Taft, 2005). Increasing a hermit word’s lexical neighbourhood density from none to one, through learning the new nonword at study (e.g., ‘ganaxy’), should therefore increase RTs in any task requiring lexical access for the associated hermit word (e.g., ‘galaxy’), relative to another hermit word that has not had a lexical neighbour studied and still has no lexical competition. Therefore, a difference in RT between these two types of words—targets (with a learned competitor) and controls (with no learned competitor)—can be used as evidence of lexical learning. This is what Coutanche and Thompson-Schill found, when measuring RTs for participants to decide whether each hermit word referred to a natural or man-made object. This evidence of lexical competition was found both 10-minutes and 24-hours after FM, but not after EE at either delay. The authors then replicated this effect in a second experiment with slightly different methods (chiefly with a reverse test order and an extended and counter-balanced stimulus set).

One minor puzzle with Coutanche and Thompson-Schill’s study is that, if lexical/semantic integration only occurs after FM, then the slowest RT in all four conditions should be for hermits with studied neighbours in the FM condition. Yet in both of their experiments, the RTs for this condition were comparable to the RTs for both hermit words with and without neighbours in the EE condition; rather it was the RTs for control words in the FM condition that were faster than the other three conditions.

Another issue concerns the question of whether FM affects lexical or semantic integration. The natural/man-made task clearly requires lexical access, but it also requires additional semantic processing (as opposed, for example, to a task like lexical decision, where a word versus non-word decision can be based on word-form only). Therefore, it is possible that FM affects this semantic processing component, rather than lexical integration per se. In Experiment 1 Coutanche and Thompson-Schill (2014) also tested performance on a semantic priming task, where they measured RTs to make a word/non-word decision about a new set of words as a function of whether an immediately-preceding word was from the same or a different semantic category (related versus unrelated). The preceding word (prime) was one of the new names learned in the FM or EE study phase. They found no evidence of faster RTs for related than unrelated primes after 10-minutes, suggesting that the newly learned names had not become semantically integrated. This result is somewhat at odds with the aforementioned evidence for ATL involvement in FM conditions, given that ATL is associated with semantic processing. Instead, Coutanche and Thompson-Schill’s findings suggest that FM only aids lexical integration.

However, a potentially more puzzling aspect is that same-day lexicalisation of new words (sufficient to affect RTs in a natural/man-made task) is rarely found in other studies on adults. While same-day lexicalisation might exist in the infant FM literature (at least based on partial knowledge, Carey & Bartlett, 1978; Swingley, 2010), lexicalisation of new words in adults usually requires consolidation through sleep, so that information acquired by the hippocampus can be integrated into longer term storage in neocortex (Bakker, Takashima, van Hell, Janzen, & McQueen, 2014; Davis, Di Betta, Macdonald, & Gaskell, 2009; Davis & Gaskell, 2009). Same-day lexicalisation of new words has been reported in adults, but only under specific circumstances (e.g., recurrent, spaced study and retrieval of new words, with interleaved repeated exposure to real-word neighbours) that encourage consolidation without sleep (Lindsay & Gaskell, 2013). Coutanche and Thompson-Schill's (2014) study suggests that lexical integration can occur within the same day using just two encoding trials, when performed as part of the FM learning procedure.

A remaining puzzle is that, when we attempted to replicate Coutanche and Thompson-Schill (2014) finding with our pictures, but with their design and stimulus ‘names’, we found no evidence of lexical competition in either the FM or EE conditions. In fact, if anything, we found evidence of the opposite pattern of lexical facilitation for hermit words with a new lexical neighbour; a finding for which we are currently testing a replication.

In a more recent study, Coutanche and Koch (2017) explored the boundaries of their lexical integration effect. They investigated whether certain participant traits (i.e, an individual’s self-reported tendency to rely on episodic, semantic or spatial memory systems in everyday life) and certain stimulus properties (i.e, the typicality of the known referent item) affect whether lexical competition is found under FM. They found lexical competition effects (slowing) under FM, assessed 10-minutes after learning, but only when 1) participants had high semantic trait scores and 2) the unknown object-'name' pairings were learned alongside more *atypical* known items. They also decomposed the original effect reported in Coutanche and Thompson-Schill (2014) and found the same dependency of the lexical competition effect on item typicality (no participant trait ratings were available in the prior study). Surprisingly however, Coutanche and Koch (2017) did not report an analysis including all participants and all items, to see whether they replicated their original evidence of lexical integration (which should have been unlikely, if the boundary conditions require both atypical semantic referents and individuals with high semantic trait scores). Indeed, further studies of FM that use implicit measures are needed to increase the evidence base.

A final puzzling aspect of Coutanche and Koch's (2017) study is the suggestion that the effect depends on using semantic referents that are atypical. An original assumption of the adult FM procedure was that activation of overlapping features is responsible for rapid cortical integration, which motivated the presentation of a known item from the same category as the unknown item (Sharon et al., 2011, even though, as reviewed earlier, this does not seem central to infant fast mapping). With Sharon et al.’s feature overlap in mind, more typical known items should activate more prominent category features that overlap with the unknown item and hence facilitate acquisition; a possibility raised in Coutanche and Thompson-Schill (2015a), and in-line with Coutanche and Thompson-Schill’s (2015b) conclusions about creation of new concepts by binding visual and semantic information. Coutanche and Koch's (2017) finding, however, suggests the opposite: greater overlap was associated with decreased lexical competition. The authors speculated that deeper semantic processes might be recruited when fewer category-typical features are present in the accompanying items, which facilitates lexical integration. Coutanche and Thompson-Schill (2015a) raise deeper encoding as a possibility of how fast cortical learning could occur, but find it not in-keeping with previous free recall results. This raises the question of whether the pattern of FM performance in implicit tasks is consistent with the theoretical framework originally proposed by Sharon et al. (2011) to explain FM performance in explicit tasks. Independent of this theoretical point, if same-day lexicalisation via FM is only possible with atypical referents and individuals with certain traits (let alone only apparent in implicit measures), then it becomes less interesting from the perspective of rehabilitation of individuals with hippocampal lesions (though it is possible that individuals with episodic memory impairment, e.g., following hippocampal lesions, might be naturally disposed towards semantic processing).

## Conclusion

The original study by Sharon et al. (2011), which could be taken to suggest that FM can ‘cure’ amnesia, has proved difficult to replicate by at least three other research groups, and thus whether FM is a way to achieve rapid, hippocampally-independent learning is still a matter of debate. In healthy adults (with an intact hippocampus), there is currently no evidence of faster or better integration of new information under FM than EE in tests of explicit memory. Additionally, the limited evidence that exists for an FM advantage in tests of implicit memory raises several additional theoretical puzzles, and deserves further replication. The question of whether fast mapping occurs in adults thus remains unresolved, much like the question of whether fast mapping is really a distinct form of learning in the developmental literature from where the concept originated.

There is some evidence from behavioural and neural (fMRI) dissociations to suggest that different processes are involved in FM and EE learning. Several puzzles remain however, such as why the components hypothesized as important for adult FM—namely the presence of a semantic referent and requirement for a semantic inference—appear to hinder rather than help explicit memory. A difficulty here is that explicit memory performance after the FM task in healthy adults may be contaminated by incidental (hippocampally-dependent) episodic encoding, and the benefits of factors that help fast mapping may be counteracted if those factors hinder episodic encoding.

How should the adult FM field move forward? One useful theoretical development, proposed by Coutanche and Thompson-Schill (2015a), might be to distinguish the neural aspects of fast mapping (e.g., whether or not rapid consolidation can occur without the hippocampus; whether fast mapping occurs in neocortex, etc.) from the cognitive aspects (e.g., whether fast mapping involves different processes to episodic encoding). For example, if the original Sharon et al. (2011) results cannot be replicated, and hippocampal damage impairs both FM and EE conditions equally, this does not invalidate the possibility that the FM condition engages lexical integration to a greater extent than the EE condition (as revealed for example by implicit measures).

In terms of useful empirical developments, one imperative would be to establish the reliability, and potential boundary conditions, of the dissociation between FM and EE conditions on implicit measures. If an FM advantage is established with implicit measures, it would then need to be reconciled with other evidence on word learning in adults, which suggest that sleep is normally required for lexical integration. Further types of implicit measure would also be needed to clarify whether the FM task affects lexical and/or semantic integration. Until such evidence is accumulated, claims that fast mapping is a special form of learning in adults remain a matter of debate.
